# Diagnostic Performance of Ovarian Morphology on Ultrasonography across Anovulatory Conditions—Impact of Body Mass Index

**DOI:** 10.3390/diagnostics13030374

**Published:** 2023-01-19

**Authors:** Heidi Vanden Brink, Brittany Y. Jarrett, Nigel Pereira, Steven D. Spandorfer, Kathy M. Hoeger, Marla E. Lujan

**Affiliations:** 1 Division of Nutritional Sciences, Cornell University, Ithaca, NY 14853, USA; 2 Department of Nutrition, Texas A&M University, College Station, TX 77840, USA; 3 Center for Reproductive Medicine, Weill Cornell Medicine, New York, NY 10021, USA; 4 Department of Obstetrics and Gynecology, University of Rochester, Rochester, NY 14620, USA

**Keywords:** ovary, diagnostic accuracy, ultrasonography, pcos, menstrual irregularity, ovarian volume, antral follicle, obesity

## Abstract

The study objectives were to determine whether ovarian morphology can distinguish between women with regular menstrual cycles, normo-androgenic anovulation (NA-Anov), and PCOS and whether body mass index (BMI)-specific thresholds improved diagnostic potential. Women with PCOS (biochemical and/or clinical hyperandrogenism and irregular cycles; N = 66), NA-Anov (irregular cycles without clinical and/or biochemical hyperandrogenism; N = 64), or regular cycles (controls; cycles every 21–35 days in the absence of clinical or biochemical hyperandrogenism; N = 51) were evaluated. Participants underwent a reproductive history, physical exam, transvaginal ultrasound, and a fasting blood sample. Linear regression analyses were used to assess the impact of BMI on ovarian morphology across groups. The diagnostic performance of ovarian morphology for anovulatory conditions, and by BMI (lean: <25 kg/m^2^; overweight: ≥25 kg/m^2^), was tested using Receiver Operating Characteristic (ROC) curves. Follicle number per ovary (FNPO) and ovarian volume (OV), but not follicle number per cross-section (FNPS), increased across controls, NA-Anov, and PCOS. Overall, FNPO had the best diagnostic performance for PCOS versus controls (AUC_ROC_ = 0.815) and NA-Anov and controls (AUC_ROC_ = 0.704), and OV to differentiate between PCOS and NA-Anov (AUC_ROC_ = 0.698). In lean women, FNPO best differentiated between PCOS and controls (AUC_ROC_ = 0.843) and PCOS versus NA-Anov (AUC_ROC_ = 0.710). FNPS better distinguished between NA-Anov and controls (AUC_ROC_ = 0.687), although diagnostic performance was lower than when thresholds were generated using all participants. In women with overweight and obesity, OV persisted as the best diagnostic feature across all analyses (PCOS versus control, AUC_ROC_ = 0.885; PCOS versus NA-Anov, AUC_ROC_ = 0.673; NA-Anov versus controls, AUC_ROC_ = 0.754). Ovarian morphology holds diagnostic potential to distinguish between NA-Anov and PCOS, with marginal differences in diagnostic potential when participants were stratified by BMI suggesting that follicle number may provide better diagnostic performance in lean women and ovarian size in those with overweight.

## 1. Introduction

Polycystic ovarian morphology (PCOM) on ultrasonography is an established marker of reproductive disturbance, most predominantly used for the diagnosis of polycystic ovary syndrome (PCOS) [1,2]. PCOM is currently defined as an upper threshold for ovarian volume (OV) and follicle number per ovary (FNPO) in healthy populations [1,2]. Therefore, the utility of ovarian imaging is limited to classifying between “normal” and “abnormal” morphology without consideration of the specificity, etiology, or potential severity of the anovulatory condition.

PCOS exists on a phenotypic spectrum with both normo-androgenic and hyperandrogenic phenotypes [1]. The normo-androgenic phenotype demonstrates ovarian dysmorphology, menstrual irregularity, and reproductive and metabolic disturbance, albeit less severe than those with the hyperandrogenic phenotypes [3,4,5,6,7]. As such, it has been hypothesized that the normo-androgenic phenotype may represent an intermediate condition which may progress to the hyperandrogenic phenotype under certain conditions (i.e., weight gain) [8,9,10]. Therefore, it is possible that clinical differentiation of normo-androgenic versus hyperandrogenic anovulatory conditions could be helpful to guide treatment strategies aimed at both treatment and prevention to avoid progression towards hyperandrogenism.

The degree of ovarian morphologic overlap between normo-androgenic and hyperandrogenic phenotypes of PCOS is unknown. Associations of follicle counts and ovarian size, with increasing androgens, gonadotropins, and intervals between menses, provide a physiological basis for the idea that features of ovarian morphology may be sufficiently different to distinguish between hyperandrogenic and normo-androgenic anovulatory conditions [11,12,13,14,15]. Given the numerous challenges in establishing reliable indicators of clinical hyperandrogenism and accessing high-quality, reliable androgen assays to assess biochemical hyperandrogenism in women [16,17], non-invasive morphological markers of anovulatory conditions may help predict comorbidity risk, refine treatment recommendations, and monitor phenotypic changes over time.

Definitions of PCOM also do not account for potential confounding effects of adiposity, despite growing evidence to support an impact of metabolic status on ovarian morphology [18]. Body weight and insulin resistance in women [15,19,20,21] and weight gain in animal models [22,23,24] have been associated with perturbations in follicle development, follicle subpopulations, and/or ovarian size. Therefore, it is plausible that adiposity and/or metabolic status impacts the specificity or sensitivity of ovarian morphology to differentiate among anovulatory conditions.

Therefore, the objectives were to first determine whether ultrasonographic features of ovarian morphology can distinguish between women with regular menstrual cycles, normo-androgenic anovulation (NA-Anov), and the hyperandrogenic phenotype of PCOS and second to determine whether consideration of BMI improved diagnostic performance.

## 2. Materials and Methods

### 2.1. Study Subjects

One hundred and eighty-one women with PCOS (N = 66), NA-Anov (N = 64), and regular menstrual cycles (N = 51) were recruited as a part of five ongoing studies (Clinicaltrials.gov identifiers: NCT01927471, NCT01927432, NCT01859663, NCT03306849, NCT01785719). Recruitment was targeted to obtain similar numbers of women categorized as controls, NA-Anov, and PCOS with a BMI < 25 kg/m^2^ (“lean”; N = 29, 35, 23, respectively) and those with a BMI ≥ 25 kg/m^2^ (“overweight”; N = 22, 29, 43, respectively). Based on a sample size calculation for a receiver operating characteristic (ROC) curve analysis in which the minimum diagnostic threshold value (AUC) of 0.80 was assigned, a sample size of at least 20 participants in each BMI sub-group would allow us to detect any significant diagnostic potential of an ovarian feature to distinguish between reproductive conditions (defined as better than chance alone; AUC = 0.50) at an α = 0.05 and β = 0.80 [25].

PCOS was defined by the NIH criteria as having both: (1) irregular menstrual cycles and (2) clinical and/or biochemical hyperandrogenism in the absence of other reasons for anovulation or androgen excess. NA-Anov was defined as the presence of irregular menstrual cycles in the absence of clinical or biochemical hyperandrogenism. Irregular menstrual cycles were defined as a history of menstrual cycles < 21 or >35 days within the past year. Clinical hyperandrogenism was defined as a modified Ferriman-Gallwey score > 6 and biochemical hyperandrogenism was defined as a total testosterone concentration > 61.5 ng/dL based on the upper 95th percentile of an internal reference population. Participants were at least 2 years post-menarche, aged 18–38 years, and had not used hormones, fertility drugs, insulin sensitizers, or drugs known to influence lipid metabolism or reproductive function for at least 2 months. Women were excluded if they were currently pregnant, nursing, had untreated abnormalities in prolactin (>25.0 ng/mL), thyroid stimulating hormone (>5.0 uIU/mL), or elevated follicle stimulating hormone levels (>20.0 mIU/mL).

### 2.2. Ethical Considerations

Studies were approved by either the Cornell University Institutional Research Board, University of Rochester Institutional Research Board, or the Weill Cornell Medicine Institutional Research Board. Interactions with participants occurred at the Human Metabolic Research Unit (Cornell University, Ithaca, NY, USA), Strong Fertility Center and Clinical Research Center (University of Rochester, Rochester, NY, USA), or the Center for Reproductive Medicine and Clinical and Translational Research Center (Weill Cornell Medicine, New York, NY, USA) between 2009 and 2018. Written informed consent was obtained before any procedures were conducted.

### 2.3. Clinical Assessments

Participants underwent the following procedures: (1) evaluation of menstrual cycle history, (2) transvaginal ultrasound, (3) fasting blood draw, (4) 2-h oral glucose tolerance test, (5) a physical exam to assess terminal hair growth using the modified Ferriman-Gallwey scoring system and (6) a vitals and anthropometry assessment (waist and hip circumference, height, weight, blood pressure). Participants were asked to fast for at least 10 h prior to the morning blood draw and oral glucose tolerance test. Hair growth on 9 regions of the body was assessed by inspection and participant self-report to generate a modified Ferriman-Gallwey hirsutism score [26]. Waist and hips circumference were measured using a tape measure and standardized procedure. Weight was collected using a calibrated digital scale and height was measured with a digital stadiometer. BMI was calculated as weight in kilograms divided by height in meters squared. Blood pressure was assessed with an automated blood pressure monitor and appropriate-sized cuff. Blood glucose was measured on-site via venous blood remaining in the butterfly line following venipuncture using a glucometer (Accu-chek Aviva, Roche) and values were used to calculate the homeostatic model assessment for insulin resistance (HOMA-IR; (Insulin0hrxGlucose0hr)/22.5) [27] and the Whole-Body Insulin Sensitivity Index (WBISI) [28].

### 2.4. Ultrasonography

All participants were scanned with a Voluson ultrasound system (GE, Milwaukee, Wisconsin) using either a RIC5-9W-RS, RIC5-9A-RS, or RIC6-12-D endo-vaginal transducer. Scans occurred in the follicular phase of participants with regular cycles (within the first 10 days from the onset of menses) or at a time when there was no dominant follicle or evidence of a recent ovulation in participants with cycle irregularity. In a subset of participants, the day of the ultrasound was different than the day of the other assessments in order to ensure the correct stage of cycle and avoid the presence of a dominant follicle or corpus luteum (Range, 1–20 days from the day of the scan). Partitioned 2D cine-loops derived from the volumes obtained during live scanning of the right and left ovaries were analyzed off-line using a grid overlay [29] by 1 of 6 members of the research team trained in image analysis to obtain FNPO. All members of the research team achieved acceptable levels of agreement (intraclass correlation coefficient (ICC) > 0.9) across their estimates of the total number of 2–9 mm follicles per ovary (FNPO) as part of an internal reliability study. The primary ovarian endpoints obtained were: (1) FNPO, (2) Follicle Number Per Cross Section (FNPS) and (3) Ovarian Volume (OV). Ovarian volume was calculated as (πx (average of four linear measurements of length and width in two orthogonal planes through sagittal and transverse views of the ovary)), which we determined to best correlate with the OV obtained via 3D ultrasonography when the true length, width, and height could be measured (unpublished data). Values reported for FNPO and OV represent the mean of measurements made in the right and left ovary for an individual participant. In cases where poor image quality prevented reliable assessments or a dominant follicle was detected in 1 of 2 ovaries, only the values of the opposite ovary were reported.

### 2.5. Biochemical Assays

Sera were assayed at the Human Nutritional Chemistry Service Laboratory at Cornell University for luteinizing hormone (LH), follicle-stimulating hormone (FSH), estradiol, insulin, and sex hormone binding globulin (SHBG) using a chemiluminescent immunoassay (Siemens Medical Solutions Diagnostics, Deerfield, IL). The intra-assay coefficients of variation (CVs) ranged from 3 to 6% and the inter-assay CVs ranged from 5 to 10%. Serum total testosterone was measured using an LC-MS/MS assay (Brigham and Women’s Hospital Research Assay Core Boston, MA), which has been certified by the Centers for Disease Control and Prevention HoST Program. The inter-assay CV was <8% and intra-assay CV was <5%. Free and bioavailable testosterone were calculated with methods described previously [30] and can also be accessed via the internet at: http://www.issam.ch/freetesto.htm (last accessed on 17 January 2023).

### 2.6. Statistical Analysis

Variables were transformed as needed to meet assumptions of normality for parametric testing. Group comparisons of sonographic, reproductive, and metabolic markers between lean and overweight cohorts within each reproductive group were assessed using one-way ANOVA or multiple linear regression. The impact of BMI was evaluated across sonographic, reproductive, and metabolic markers using linear regression analyses where BMI, phenotype, and an interaction effect were tested. Multiple comparisons were conducted using Tukey’s HSD test. The accuracy of sonographic markers to diagnose NA-Anov and PCOS versus controls and NA-Anov from PCOS were determined using Receiver Operating Characteristic (ROC) curves. Diagnostic thresholds were proposed based on Youden’s index, which balances maximum test specificity and test sensitivity. Comparisons of diagnostic accuracy were conducted across groups qualitatively, by contrasting AUC_ROC_, specificity, and sensitivity. Partial correlations were conducted to assess the independent associations between diagnostic features of anovulatory conditions and BMI. Statistical analyses were conducted in JMP Pro (version 16.0) and MedCalc (version 20). Significance was defined as *p* < 0.05.

## 3. Results

### 3.1. Characteristics of Participants across Reproductive Phenotypes

Demographic, diagnostic, metabolic, and reproductive features of the participants are presented in Table 1. BMI and age were different across cohorts (Table 1), therefore multiple linear regression analyses were conducted to contrast features adjusting for differences in age and BMI across cohorts. By design, clinical and biochemical measures of androgen status were lower in controls and NA-Anov versus PCOS (Table 1). Likewise, menstrual cycle length was increased in both anovulatory groups versus controls. Women with NA-Anov and PCOS had increased 2-h insulin following the OGTT versus controls, but only women with PCOS were more insulin resistant, as defined by the WBISI, versus controls. Last, FNPO and OV, but not FNPS, increased in a stepwise fashion from control to NA-Anov to PCOS.

### 3.2. Diagnostic Potential of Ovarian Morphology across Reproductive Phenotypes

**Figure 1:**
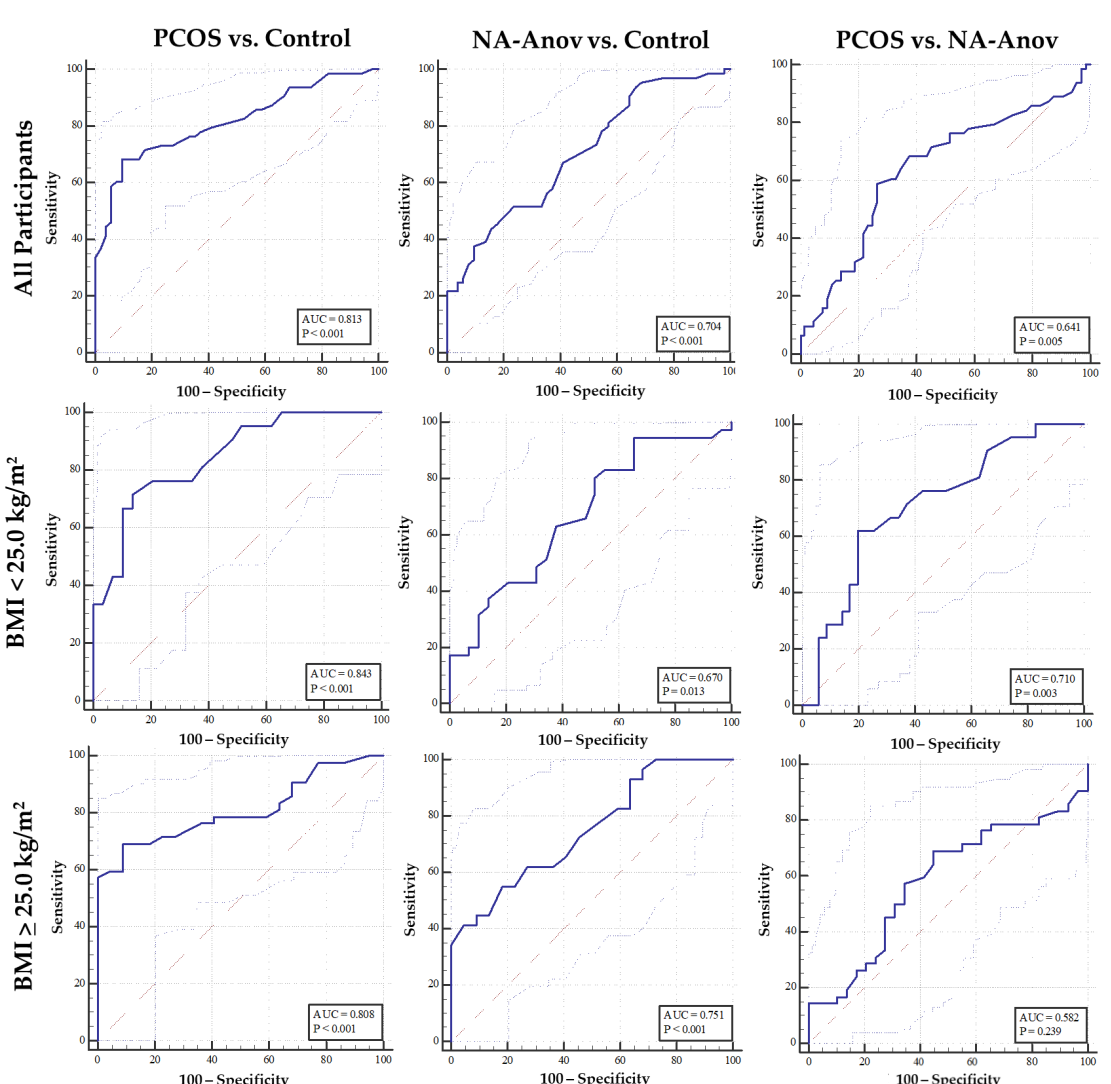
Diagnostic accuracy of FNPO 2-9mm for anovulatory conditions among all participants (**Top Row**), lean participants (**Middle Row**), and participants with overweight or obesity (**Bottom Row**).

The diagnostic ability of each sonographic marker to distinguish between reproductive phenotypes is presented in Table 2. ROC curves demonstrating the diagnostic performance of FNPO are presented in Figure 1 (top row). FNPO, FNPS, and OV all had significant diagnostic potential to discern PCOS from controls (*p* < 0.001) at thresholds of 35 follicles, 8 follicles, and 8.2 cm^3^, respectively. FNPO, FNPS, and OV also exhibited significant diagnostic potential to discern NA-Anov from controls (*p* < 0.001) at thresholds of 33 follicles, 8 follicles, and 5.7 cm^3^, respectively. By contrast, FNPO and OV, but not FNPS, exhibited diagnostic potential for PCOS versus NA-Anov at thresholds of 39 follicles and 8.1 cm^3^ (*p* = 0.0051 and *p* < 0.0001, respectively). In the case of both FNPO and OV, the diagnostic performance of these ovarian features for PCOS versus NA-Anov was poorer as reflected in lower AUC_ROC_ values and reduced test specificity.

### 3.3. Impact of Body Mass Index on Diagnostic Potential of Ovarian Morphology across Reproductive Phenotypes

Table 3 summarizes the performance of FNPS, FNPO and OV as diagnostic markers for anovulatory conditions in lean and overweight groups. ROC curves demonstrating the diagnostic performance of FNPO by BMI cohort are also presented in Figure 1 (middle and bottom row). FNPO, FNPS, and OV had significant diagnostic potential to distinguish between PCOS and controls when lean and overweight women were considered separately. Stratification by BMI-status showed that FNPO had greater diagnostic accuracy in lean women (AUC_ROC_ = 0.843), whereas OV had greater diagnostic accuracy in the overweight cohort (AUC_ROC_ = 0.885), when compared to analyses involving all participants combined (FNPO AUC_ROC_ = 0.815 and OV AUC_ROC_ = 0.830, Table 2).

When all participants were considered together, FNPO exhibited the greatest specificity and discriminatory power between NA-Anov and controls at threshold of 33 (specificity, 84% AUC_ROC_ = 0.704), albeit the lowest sensitivity (44%) versus FNPS and OV. Follicle counts (FNPS and FNPO) had diagnostic accuracy to discriminate between NA-Anov and controls in both lean and overweight groups. However, diagnostic accuracy in lean women was not improved when stratified by BMI. In the case of OV, this marker only had diagnostic potential for NA-Anov in the overweight group, demonstrating slightly higher diagnostic accuracy compared to markers of follicle excess and superior to thresholds generated when all participants were considered together.

FNPS did not exhibit any diagnostic potential to distinguish between PCOS and NA-Anov when lean and overweight participants were considered separately (Table 3) or combined (Table 2). By contrast, FNPO had diagnostic potential to distinguish between PCOS and NA-Anov in lean women and with slightly improved diagnostic accuracy compared to when all participants were combined (AUC_ROC_ 0.710 versus 0.641, respectively; Table 2). Finally, OV distinguished between anovulatory conditions when women were separated by BMI category. However, diagnostic accuracy was lower for lean (OV AUC_ROC_ = 0.684) and overweight (OV AUC_ROC_ = 0.673) groups when compared to analyses involving all participants combined (OV AUC_ROC_ = 0.698, Table 2).

## 4. Discussion

In the present study, we tested the hypothesis that ultrasonographic assessments of the ovary could distinguish across anovulatory conditions and eumenorrhea along a BMI spectrum. In partial support of our hypothesis, we found that the diagnostic performance of FNPO, FNPS, and OV differed depending on the anovulatory phenotype and was additionally impacted by BMI status.

FNPO exhibited diagnostic potential to discriminate between PCOS and NA-Anov with marginal improvements when the overweight cohort was excluded. This analysis represents the clinical scenario in which a patient presents with menstrual irregularity and the clinician seeks to differentiate between a hyperandrogenic versus normo-androgenic phenotype. The loss of discriminatory power, sensitivity, and specificity suggest marginal potential of FNPO to discriminate between PCOS and NA-Anov. We had anticipated a more robust association attributed to pronounced follicular excess between PCOS and NA-Anov and FNPO or OV, given that antral follicles are the central source of testosterone leading to hyperandrogenism [33] and hyperandrogenism is capable of disrupting folliculogenesis. The degree of morphologic overlap between the two groups may be attributed to inclusion of hirsutism in our clinical definition of hyperandrogenism in PCOS. Several studies have failed to demonstrate an association between hirsutism and total androgen production [34,35,36,37], suggesting that hirsutism may not be an adequate biomarker of current ovarian androgen production. Therefore, inclusion of hirsutism as evidence for hyperandrogenism may have decreased the magnitude of effect between PCOS and the NA-Anov phenotypes. Increased follicle number is also independently associated with both menstrual cycle disturbances and hyperandrogenism [11,12,13,14,15], therefore its plausible that disruptions in ovarian folliculogenesis associated with menstrual irregularity impaired our ability to detect differences in ovarian morphology attributed to androgen status alone. How or whether mechanisms of anovulation and hyperandrogenism are synergistic or additive in their influence on folliculogenesis and ovarian morphology are unknown. Ultimately, these data suggest that hyperandrogenism induces insufficient differences in follicle number to yield FNPO as a sufficiently robust marker in the context of anovulation.

We also report diagnostic potential for FNPO and OV at a substantially different thresholds than the International Guidelines of 20 follicles (FNPO) and 10 cm^3^ (OV) [1]. While our results align with previous diagnostic studies establishing the diagnostic potential of FNPO to discriminate between PCOS and controls [12,31,38,39,40], we report higher FNPO (35 follicles vs. 12 [12], 19 [38], and 26 [31] follicles) and lower OV (7–8 cm^3^ [38,39,41,42,43,44]) thresholds. It is likely that our off-line approaches to image analysis resulted in higher follicle counts [29] but were unlikely to influence OV. Difference in thresholds may also be attributed to our *a priori* intention to have similar BMI cohorts between controls and anovulatory cohorts; control cohorts in the previous studies were within a healthy BMI range (<25 kg/m^2^, mean), whereas PCOS participants had, on average, BMIs within overweight or obese categories [12,31,38]. Because metabolic status is believed to influence ovarian physiology [18], we designed this study to account for the contribution of adiposity to differences in ovarian morphology.

Ovarian morphology exhibited low specificity to distinguish between NA-Anov and controls, implying that eumenorrheic individuals have a broad range of FNPO which overlaps with oligo- or amenorrhea in the absence of androgen excess. Previous studies evaluating a normo-androgenic phenotype of PCOS use a pre-specified diagnostic threshold to define their cohort, which excludes participants with NA-Anov whose FNPO overlap with the control cohort. Similarly, the control cohort often excludes eumenorrheic women with increased follicle numbers [38,45]. Because we did not use ovarian morphology as an inclusion or exclusion factor in the present study, we consider the degree of overlap noted to be representative of the true variance that one would expect to encounter when evaluating women with anovulatory conditions.

We had anticipated that obesity (and/or its metabolic consequences) would influence follicle dynamics sufficiently to impact ovarian morphology and alter its specificity and sensitivity for anovulatory conditions. In partial support of our hypothesis, we report evidence of marginally improved discriminatory power of ovarian morphology when women were stratified by lean and overweight categories. In the absence of androgen excess, a lower FNPO was needed to discriminate lean women with irregular cycles from controls (>16 follicles) versus their overweight counterparts (>33 follicles). For the diagnosis of PCOS, FNPO had superior diagnostic potential for PCOS versus both controls and NA-Anov in the lean cohort (>32 and >29 follicles, respectively), whereas OV exhibited the best diagnostic potential for PCOS in the overweight group versus both controls and NA-Anov (>7.1 and 7.5 cm^3^, respectively). These data suggest that physiological mechanisms related to obesity may influence folliculogenesis, but not ovarian size, thereby compromising the diagnostic potential of FNPO. There is a biological premise for this assertion in pre-clinical models. Studies in non-human primates consuming a western-style, high fat diet showed the development of an increased number of small follicles in conjunction with reduced LH pulse frequency [22,24,46]. Rodent studies have also demonstrated that diet-induced obesity in the absence of hyperandrogenism is associated with accelerated follicle development [23,47], and increased follicle populations [48,49]. That WSD alone could induce a polycystic-like ovarian morphology may explain why FNPO did not significantly discriminate between anovulatory groups among women with a BMI ≥ 25 kg/m^2^. Overall, our data point to the possibility that thresholds which consider metabolic status in some way may be needed to maintain diagnostic accuracy of ovarian features for anovulatory disorders.

This study had several strengths. We used reliable methods to assess ovarian morphology that enabled us to draw a reliable conclusion regarding the diagnostic potential of the ovary for anovulatory disorders. The sonographic, metabolic, and reproductive measures in this study are typical of a clinical evaluation and therefore provide a measure of translational utility for this research. Our use of a nationally standardized program to obtain reliable measures of testosterone ensures the capacity to corroborate findings across studies. We also acknowledge the limitations of this study. This study was not designed to consider the relative sources of androgen production which has implications for understanding the true etiology of anovulatory conditions. Studies have confirmed that the ovaries are a key source of testosterone over-production in women with PCOS [33]. The extent to which adrenal versus ovarian androgens impact reproductive and metabolic status is uncertain [50]. We also acknowledge that BMI represents an imperfect measure of adiposity and metabolic status and therefore may not capture the mechanisms underlying improvements in diagnostic accuracy. Finally, our definition of hyperandrogenism differs from the 2018 guideline for the diagnosis of PCOS [1]. This study was designed before the release of the guideline, and therefore reflects an effort to test our a priori hypotheses. However, it is noteworthy that, under these newest definitions of hyperandrogenism, a large subset of women with NA-Anov in this study would be phenotyped as hyperandrogenic PCOS given the finding of an isolated elevation in free testosterone

This study corroborated the diagnostic potential of sonographic aspects of ovarian morphology to detect anovulatory disorders. Overlap in follicle populations across anovulatory conditions limited its specificity for the etiology of anovulation. Consideration of adiposity, or its metabolic consequences, on ovarian morphological features of anovulation may be helpful in refining diagnostic thresholds. By improving our understanding of relationships between metabolic disturbances and the reproductive axis, we may have the potential to identify diagnostic features unique to specific etiologies of reproductive disturbances.

## Figures and Tables

**Table 1:** Demographic, Diagnostic, Metabolic, and Reproductive Features of all Women Across Cohorts.

	Controls		NA-Anov		PCOS	
	Mean	SD	Mean	SD	Mean	SD
Demographics						
N	51		64		66	
Age (y)	28	5.4 ^a^	26	5.4 ^b^	26	5.3 ^b^
Age at menarche (y)	12	1.3	13	1.4	13	1.8
Ethnicity (N, (%))						
Hispanic or Latino	6	(12)	6	(9)	6	(9)
Not Hispanic or Latino	36	(70)	51	(80)	55	(83)
Other or Not Reported	9	(18)	7	(11)	5	(8)
Race (N, (%))						
White	33	(65)	41	(64)	44	(66)
Black or African American	5	(10)	6	(9)	8	(12)
Asian	3	(5)	13	(20)	7	(11)
Native Hawaiian or Other Pacific Islander	0	(0)	0	(0)	0	(0)
More than one race	5	(10)	1	(2)	2	(3)
Other or Not Reported	5	(10)	3	(5)	5	(8)
Diagnostic Features						
Menstrual Cycle Length (d)	29	2.2 ^a^	72	63.8 ^b^	98	105.5 ^b^
Hirsutism Score	3	2.1 ^a^	3	1.8 ^a^	10	5.2 ^b^
Total T (ng/dL)	29.0	14.47 ^a^	33.7	15.58 ^a^	52.3	27.43 ^b^
Metabolic Status						
BMI (kg/m^2^)	27.1	6.7 ^a^	28.2	9.0 ^a,b^	31.2	9.3 ^b^
WHR	0.83	0.06	0.81	0.08	0.85	0.09
Fasting Glucose (mg/dL)	94.9	9.41	92.7	9.99	96.0	12.90
Fasting Insulin (uIU/mL)	7.8	6.11	9.5	8.27	13.9	12.63
2-HR Glucose	93.5	26.43	93.4	20.22	105.0	34.61
2-HR Insulin	33.4	30.75 ^a^	54.4	37.93 ^b^	83.1	87.72 ^b^
HOMA-IR	1.8	1.50	2.2	1.97	3.5	3.56
WBISI	10.4	6.68 ^a^	7.9	6.34 ^a,b^	6.2	5.39 ^b^
Reproductive Endocrinology						
LH (mIU/mL)	4.7	2.33 ^a^	7.6	5.15 ^a,b^	7.9	3.48 ^b^
FSH (mIU/mL)	6.5	2.20	6.0	2.06	6.1	1.73
Estradiol (pg/mL)	55.7	38.55	54.9	32.96	58.3	42.25
SHBG (nmol/L)	56.8	25.88	57.2	42.39	42.2	25.09
Free T (ng/dL)	0.4	0.19 ^a^	0.5	0.33 ^a^	0.9	0.56 ^b^
Bioavailable T (ng/dL)	8.9	4.47 ^a^	12.1	7.68 ^a^	21.3	13.12 ^b^
FAI (%)	2.1	1.61 ^a^	3.1	2.59 ^a^	6.1	5.18 ^b^
Ovarian Morphology						
FNPS	7	3.3 ^a^	9	4.2 ^b^	10	4.4 ^b^
FNPO 2–9 mm	24	10.2 ^a^	35	16.5 ^b^	45	24.0 ^c^
OV (cm^3^)	6	2.2 ^a^	8	3.3 ^b^	10	3.5 ^c^

Overall *p* values are not reported but reflect analyses conducted using raw or transformed data to meet assumptions, adjusting for age and BMI. When the overall *p* was significant, post hoc between-group differences were conducted using Tukey’s HSD. Within each row, significant differences between phenotypes are denoted by different superscript letters following post-hoc analyses. Statistical analyses were not conducted for race and ethnicity across reproductive cohort and therefore only descriptives are reported. Abbreviations include: T, Testosterone; BMI, Body Mass Index; HOMA-IR, Homeostatic Model Assessment of Insulin Resistance; WHR, Waist-Hip Ratio; WBISI, Whole Body Insulin Sensitivity Index; LH, Luteinizing Hormone; FSH, Follicle Stimulating Hormone; FAI, Free Androgen Index; FNPO, Follicle Number Per Ovary; OV, Ovarian Volume. For SHBG, to convert from SI units (nmol/L) to conventional units (mg/dL), divide by 10.

**Table 2:** Diagnostic Potential of Ovarian Morphology to Distinguish between Anovulatory Conditions.

	Threshold	AUC_ROC_	*p* Value	Sensitivity (%)	Specificity (%)
PCOS vs. Control					
FNPS	>8	0.753	<0.0001	71 (58–82)	71 (56–83)
	>9 [31]			58 (46–71)	77 (63–87)
	>10 [32]			48 (35–61)	86 (74–94)
FNPO	>35	0.813	<0.0001	68 (55–79)	90 (77–97)
	>20 [2]			87 (77–94)	37 (24–52)
	>25 [1]			83 (71–91)	47 (33–62)
OV (cm^3^)	>8.2	0.830	<0.0001	73 (60–83)	84 (71–94)
	>10 [1,31]			42 (30–55)	94 (84–99)
NA-Anov vs. Control					
FNPS	>8	0.685	<0.0001	56 (43–69)	70 (56–83)
	>9			50 (37–63)	74 (63–87)
	>10			37 (26–51)	86 (74–94)
FNPO	>33	0.704	<0.0001	44 (31–57)	84 (71–93)
	>20			86 (75–93)	37 (24–52)
	>25			73 (61–83)	47 (33–62)
OV (cm^3^)	>5.7	0.656	0.0023	75 (63–85)	55 (40–69)
	>10			17 (9–29)	94 (84–99)
PCOS vs. NA-Anov					
FNPS	>7	0.570	0.1667	78 (67–88)	38 (26–51)
	>9			58 (46–71)	50 (37–63)
	>10			48 (35–61)	63 (50–74)
FNPO	>39	0.641	0.0051	59 (46–71)	73 (61–84)
	>20			87 (77–94)	14 (7–25)
	>25			83 (71–91)	27 (16–39)
OV (cm^3^)	>8.1	0.698	<0.0001	74 (62–84)	69 (56–80)
	>10			42 (30–55)	80 (68–89)

Diagnostic potential as judged by area under the receiver operating characteristic (ROC) curve is shown. Youden’s Index was used to identify the threshold value to balance between test sensitivity and test specificity. Abbreviations include: AUC_ROC_, Area Under the ROC Curve; FNPO, Follicle Number Per Ovary; Lean, representing participants with a BMI < 25 kg/m^2^; OW, overweight, representing participants with a BMI > 25 kg/m^2^; OV, Ovarian Volume; CI, 95th percentile confidence interval. Diagnostic performance of previously published thresholds using the present dataset are included for: Lujan et al., 2013 [31]; Allemand et al., 2006 [32]; Dewailly et al., 2014 [2]; Teede et al., 2018 [1].

**Table 3:** Diagnostic Potential of Ovarian Morphology to Discriminate between Anovulatory Conditions and Controls by Body Mass Index Category.

	Threshold	AUC_ROC_	*p* Value	Sensitivity (%)	Specificity(%)
HA-PCOS vs. Control					
FNPS					
Lean	>7	0.806	<0.001	100 (85–100)	58 (39–77)
OW	>10	0.762	<0.001	47 (31–62)	100 (85–100)
FNPO					
Lean	>32	0.843	<0.001	71 (48–89)	87 (69–96)
OW	>35	0.808	<0.001	69 (53–83)	91 (71–99)
OV					
Lean	>7.9	0.763	<0.001	65 (43–84)	79 (60–92)
OW	>7.1	0.885	<0.001	84 (69–93)	86 (65–97)
NA-Anov vs. Control					
FNPS					
Lean	>8	0.687	0.005	63 (45–77)	69 (49–84)
OW	>10	0.679	0.017	34 (18–955	100 (85–75)
FNPO					
Lean	>16	0.670	0.013	94 (81–99)	35 (18–55)
OW	>33	0.751	<0.001	55 (36–74)	81 (60–95)
OV					
Lean	>5.6	0.580	0.275	69 (51–83)	55 (36–74)
OW	>7.1	0.754	<0.001	62 (42–79)	86 (65–97)
HA-PCOS vs. NA-Anov					
FNPS					
Lean	>7	0.607	0.151	100 (85–100)	29 (15–46)
OW	>7	0.574	0.293	67 (51–80)	48 (29–68)
FNPO					
Lean	>39	0.710	0.003	62 (38–82)	80 (63–92)
OW	>34	0.582	0.239	69 (53–82)	55 (36–74)
OV					
Lean	>8.1	0.684	0.014	65 (43–84)	77 (60–90)
OW	>7.5	0.673	0.010	81 (67–92)	58 (39–77)

Diagnostic potential as judged by area under the receiver operating characteristic (ROC) curve is shown. Youden’s Index was used to identify the threshold value to balance between test sensitivity and test specificity. Abbreviations include: AUC_ROC_, Area Under the ROC Curve; FNPO, Follicle Number Per Ovary; Lean, representing participants with a BMI < 25 kg/m^2^; OV, Ovarian Volume; OW, overweight, representing participants with a BMI ≥ 25 kg/m^2^.

## Data Availability

The data that support the findings of this study are available from the corresponding author upon reasonable request.
